# Identification and Characterization of Genes Involved in *Leishmania* Pathogenesis: The Potential for Drug Target Selection

**DOI:** 10.4061/2011/428486

**Published:** 2011-06-26

**Authors:** Robert Duncan, Sreenivas Gannavaram, Ranadhir Dey, Alain Debrabant, Ines Lakhal-Naouar, Hira L. Nakhasi

**Affiliations:** Division of Emerging and Transfusion Transmitted Diseases, Center for Biologics Evaluation and Research, FDA, Bethesda, MD 20852, USA

## Abstract

Identifying and characterizing *Leishmania donovani* genes and the proteins they encode for their role in pathogenesis can reveal the value of this approach for finding new drug targets. Effective drug targets are likely to be proteins differentially expressed or required in the amastigote life cycle stage found in the patient. Several examples and their potential for chemotherapeutic disruption are presented. A pathway nearly ubiquitous in living cells targeted by anticancer drugs, the ubiquitin system, is examined. New findings in ubiquitin and ubiquitin-like modifiers in *Leishmania* show how disruption of those pathways could point to additional drug targets. The programmed cell death pathway, now recognized among protozoan parasites, is reviewed for some of its components and evidence that suggests they could be targeted for antiparasitic drug therapy. Finally, the endoplasmic reticulum quality control system is involved in secretion of many virulence factors. How disruptions in this pathway reduce virulence as evidence for potential drug targets is presented.

## 1. Introduction

*Leishmania* is the causative agent of leishmaniasis, a spectrum of diseases affecting more than 12 million people worldwide. The two major clinical forms of leishmaniasis, cutaneous and visceral, are the result of infection by different species of the parasite. Visceral leishmaniasis (VL), which causes splenomegaly and hepatomegaly, is fatal if not treated and is caused by *L. donovani* and *L. infantum *(also designated* L. chagasi *in the new world). More than 90% of the visceral cases in the world are reported from Bangladesh, India, Nepal, Sudan, and Brazil [1]. Cutaneous leishmaniasis (CL) causes lesions which are mostly self-healing and are caused by *L. major*, *L. tropica* or *L. aethiopica*, in the old world and by *L. mexicana* or the *L. braziliensis* complex in the new world [2]. Both environmental risk factors such as massive displacement of populations, urbanization, deforestation, and new irrigation plans and individual risk factors such as HIV, malnutrition, and genetic susceptibility make leishmaniasis an important public health problem [1]. Though the most significant public health effects of leishmaniasis are concentrated in developing countries, occasional cases occur in developed countries as well. In the European countries around the Mediterranean basin and throughout the Middle East, as well as Latin America, there are large populations that must still consider the risk of leishmaniasis. In some of these countries, dogs represent an important reservoir for the parasite. In the USA, even though leishmaniasis is not endemic, infections can be found in pockets of the country especially in the southwest [3]. In addition, *Leishmania* infection was found in dogs in the northeastern part of the USA [4]. Increasing immigration, tourism, and military activity in *Leishmania* endemic areas has led to leishmaniasis becoming an increasing threat in nonendemic areas of the world. This was underscored by the recent US military deployments to *Leishmania* endemic areas such as Iraq and Afghanistan, which have resulted in infected US soldiers [5]. In addition, there have been several documented cases of parasite transmission by blood transfusion worldwide forcing the deferral of exposed individuals from blood donation [6]. Studies in animal models, such as hamsters and dogs, show that *Leishmania* not only survives blood-banking storage conditions, but also retains its infectivity [7, 8]. Therefore, *Leishmania* has a potential to impact blood safety in developed as well as developing countries.

In the *Leishmania* life cycle, the motile promastigote form that resides in the alimentary canal of the sandfly vector is transmitted to a mammalian host during a blood meal. Host macrophages ingest the parasites, which must differentiate into the nonmotile, amastigote, form to persist in the macrophage's lysosomal compartment [9]. These two life stages have been adapted to *in vitro* culture for many *Leishmania* species [10, 11] allowing manipulation of the genome and assessment of the altered phenotypes *in vitro* [12, 13].

The only available cure for visceral leishmaniasis is drug treatment. Though most cutaneous leishmaniases are self-healing, drug treatment is employed to relieve the painful sores, avoid scarring and other complications. However, currently available drugs for leishmaniasis are far from satisfactory because they are toxic, expensive or lose effectiveness due to the development of drug resistance after prolonged use [14–16]. Vaccination is not a viable option either, because there are as yet no effective vaccines for leishmaniasis. Recent technological advances in the understanding of the pathogenesis of leishmaniasis beg the question how these advances could be translated into either development of better drug or vaccination strategies that could eradicate this disease.

Many investigators in the field have pointed to the importance of the publically available DNA sequence for *Trypanosomatid* genomes as a pathway to new drug discovery [17, 18]. However, for the visceral genome sequenced, *L. infantum*, there are 8387 genes annotated, of which 5,342 are “hypothetical” and only 3,288 have been assigned gene ontology terms. Thus the majority of the building blocks of this parasite are uncharacterized. A similar situation exists for the cutaneous species, *L. major*, with 5,396 hypothetical genes out of 9,388 annotated. Clearly to make advances in the development of new drugs, parasite components that are required for survival need to be identified and characterized to the point where rational drug design can target inactivation of these molecules or their activities. The annotated genome information is essential in the process of identifying and characterizing parasite proteins and the genes that encode them. Therefore, further characterization of such genes is needed to focus on the following important questions, for example: (a) how essential is a protein encoded by such genes for survival of the parasite, (b) what functional role does it play in the parasite's physiology, (c) how does it fit into biochemical pathways that are crucial for parasite pathogenesis, (d) are there life cycle stage-specific expression patterns, in particular, is the protein required in the amastigote stage that will be subject to the drug impact in treated patients, (e) how divergent is the parasite protein or activity from similar human proteins to avoid toxicity of any proposed drug, and (f) have the activities of similar proteins been inhibited with compounds that suggest drug treatment is feasible?

This paper focuses on our efforts to identify and characterize *Leishmania donovani* genes and the proteins they encode for their role in pathogenesis. A brief survey of those proteins and their novel attributes can reveal the value of this approach for finding new drug targets and illustrate specific characteristics that could suggest a target is “druggable.” We are indeed cognizant of the efforts by other investigators in this field, but have not attempted to cover those studies because of the limited scope of the paper. The search for such proteins and activities in these human pathogens requires a broad perspective on the physiology of the parasite. We present below a survey that spans diverse pathways with potential for therapeutic disruption. Any pathway that is to be targeted by drugs given to the mammalian host must be essential in the amastigote life cycle stage found in the patient. We review some examples of newly described proteins and their pathways that are differentially expressed or required in this intracellular stage in the first section. A pathway nearly ubiquitous in living cells already has been targeted by anticancer drugs, the ubiquitin system. Section two reviews new findings in ubiquitin and ubiquitin-like modifiers in *Leishmania* and how disruption of those pathways could reduce the viability of the parasite. The existence of a programmed cell death pathway has been well documented in protozoan parasites. We review some of the components of this pathway and evidence that suggests they could be targeted for drug therapy in Section three. At the very inception of synthesis of many secreted virulence factors is the endoplasmic reticulum quality control system. How disruptions in this pathway reduce virulence as evidence for a potential drug target is presented in Section four.

## 2. Targeting Proteins Uniquely Required for Survival in the Mammalian-Infecting, Amastigote, Life Cycle Stage

In search of functions that may be unique to amastigotes, we noted that the shift of metabolism from promastigotes to amastigotes leads to the expression of a spectrum of genes that could be targets to control *Leishmania* pathogenesis. Whereas promastigotes utilize glucose as their primary energy source, intracellular amastigotes depend primarily on amino acids and fatty acids as their carbon source [19, 20]. Increased mitochondrial activity may play a crucial role in the survival of amastigotes inside host cells [20, 21]. The mitochondrion harnesses the energy from numerous substrates through the electron transport chain. Electron transport depends on multiprotein complexes I, II, III, and IV embedded in the inner mitochondrial membrane ultimately passing the electron to oxygen. This oxygen consumption is referred to as respiration. The proton gradient produced by electron transport drives the F_1_/F_0_ ATPase (complex V) in a coupled process termed oxidative phosphorylation. Active respiration is required for survival of both promastigote and amastigote forms of *Leishmania *[22, 23]. Investigations of the individual complexes of the respiratory chain suggest NADH dehydrogenase (complex I) is not found in its classical form in trypanosomatids [24]. However, evidence for succinate dehydrogenase (complex II), cytochrome c reductase (complex III), and cytochrome c oxidase (complex IV) has been demonstrated for both *Leishmania *and* Trypanosoma *[24, 25]. Recent studies suggest that *Leishmania* cytochrome c oxidase is a potential target for the oral drug, Miltefosine [26, 27]. The trypanosomatid cytochrome c oxidase (COX) complex (complex IV) is a multicomponent complex composed of more than 14 subunits [28, 29]. It has three mitochondrially encoded subunits, and all the others are nuclear encoded subunits. Most of the nuclear encoded components have no apparent homologue outside the *Trypanosomatids *[28, 30] thus fulfilling one of the criteria of a drug target. Some of the nuclear encoded subunits are essential for proper function of complex IV [31] including the recently described MIX protein [32, 33]. 

Recently, we characterized a gene encoding a 27 kDa mitochondrial membrane protein (Ldp27), a subunit of the active COX complex, specific to amastigotes and metacyclics, the infectious stages in* Leishmania *[34]. We also demonstrated that Ldp27 is necessary for the high level of COX activity in amastigotes and that Ldp27 gene deleted parasites (Ldp27^−/−^) show significantly less COX activity and reduced ATP synthesis in intracellular amastigotes compared to wild type. Moreover, the Ldp27^−/−^ parasites are less virulent both in human macrophages and in BALB/c mice.

A functional role for Ldp27 is also suggested by the lower level of COX activity in the wild-type procyclic promastigote stage that does not express Ldp27. It has been established that the respiratory chain is active in *Leishmania* promastigotes [24], and the inhibition of promastigote proliferation by cyanide indicates the requirement for an active COX in this stage [23]. In our recent study, COX activity was also detected in the promastigote form, although significantly less than in the amastigote form. Thus Ldp27 may play a role in increasing the enzymatic activity of the COX complex, but not in the abundance or assembly of at least some of its components.

The utility of the electron transport chain as a target of antiparasitic drugs is illustrated by the ability of atovaquone to block growth of *Plasmodium *[35], and inhibition of the cytochrome c oxidase complex in particular is the mode of action of the antimalarials artesunate [36] and artemisinin [37]. Further study will be required to determine what specific function allows Ldp27 to substantially increase COX activity potentially through evaluation of the effect of mutating key amino acid residues. However, from the investigation so far, this protein is essential in the amastigote stage, is demonstrated to be in a critical biochemical pathway that is already known to be an effective drug target, and is a unique parasite protein suggesting specific inhibitors will not affect mammalian COX activity. These features illustrate how careful characterization of parasite proteins can set the stage for rational drug design.

In our efforts to identify genes that are differentially expressed in the virulent amastigote stage of the parasite, we identified a* Leishmania* homologue of the mammalian argininosuccinate synthase (ASS) gene first identified in a screen for genes altered in expression when amastigote cells undergo mitotic arrest. The ASS gene was also shown to be more abundantly expressed in the amastigotes than in the promastigote forms by Northern and Western blot analyses [38]. Thus this protein presents as an available target in the human infection for drug intervention.

Mammalian ASS, 59.6% similar to *Leishmania* ASS, is the limiting enzyme of the urea cycle that catalyses the ATP-dependent condensation of citrulline and aspartate to form argininosuccinate, immediate precursor of arginine, thus leading to the production of urea in the liver and Nitric Oxide (NO) in many other cells [39]. Though the high level of similarity raises early concerns about drugs having a toxic effect on the human cells as well as *Leishmania*, the subcellular compartmentalization of the protein may lead to differential sensitivity. The intracellular ASS location in mammals may depend on its physiological function, and its gene regulation differs greatly depending on the tissue [40]. Unlike the mammalian homologue, the *Leishmania* ASS is isolated to a glycosome-like vesicle, which might suggest a drug effect that differs between *Leishmania *and humans. The glycosomal localization is suggested by the glycosomal targeting signal (amino acids Serine-Serine-Leucine) encoded at the C-terminal of the amino acid sequence [41]. Further evidence comes from IFA studies using parasites overexpressing ASS with a native C terminus or ASS for which the SSL at the C-terminus was blocked with an epitope tag. The native ASS is localized in small punctate spots distributed throughout the cell, but the protein with the C terminal tag remained in the cytosol in the parasite and did not target to any cytoplasmic vesicle [42]. The unique compartmentalization in glycosomes has been suggested as a means to develop *Leishmania-*specific inhibitors of other metabolic enzymes as well [43]. An added advantage of characterization of ASS as drug target is the availability of inhibitors already used in other species that could be evaluated for anti-*Leishmania* activity. Fumonisin B1, a fungal mycotoxin altering sphingolipid metabolism through interruption of de novo ceramide synthesis, inhibits *in vitro* argininosuccinate synthase [44]. *㸁*Saccharopine, another ASS inhibitor, is a potent inhibitor of crude and purified preparations of argininosuccinate synthase [45]. Though these inhibitors may be toxic or nondiscriminating in their current form, they could be a starting point to screen chemical derivatives with improved properties. The criterion that ASS should be an essential activity has not been fully demonstrated. More characterization such as the demonstration that ASS is essential for survival as an amastigote and the description of subsequent steps in its metabolic pathway since the parasite does not have the enzymes to convert argininosuccinate to arginine will be needed; however the increased expression in the amastigote stage, the important biochemical pathway, the existence of specific inhibitors, and the divergence in subcellular localization between the mammalian enzyme and the *Leishmania *enzyme indicate a potential for ASS as a target of therapeutic drugs to treat leishmaniasis.

## 3. The Ubiquitin Conjugation System as Target for Chemotherapy

Covalent attachment of ubiquitin (Ub) to protein targets has been recognized as an important step in the specific destruction of proteins in the proteasome [46]. On the other hand, a broad range of physiological processes are regulated by an expanding array of Ub-like modifiers (NEDD, SUMO, Ufm1). The Ub/Ubl modifiers share a structural fold and are probably evolved from prokaryotic sulphurtransferase systems [47]. Ubiquitin, a 76-amino-acid protein, is covalently linked to lysine residues of substrate proteins in a multistep process. Such ubiquitination is common in normal, as well as pathological, cellular processes. The concept that ubiquitination is solely the process that targets proteins for degradation by the proteasome has been rendered over simplistic by the discovery of expanding functions regulated by ubiquitination such as protein trafficking, the assembly of protein signaling complexes, cellular remodeling through autophagy, and the activation or inactivation of enzymes [47]. The attachment of Ub to a substrate requires the consecutive action of three enzymes. The first step involves the activation of Ub by the formation of a thioester bond with the ubiquitin-activating enzyme, E1. In the second step, E1 delivers the activated Ub to the E2 ubiquitin-conjugating enzyme. Finally, E3 ligases catalyse the transfer of Ub from E2 to a lysine residue in the substrate protein. Ubiquitin contains seven acceptor lysines that can be conjugated with ubiquitin, giving rise to ubiquitin chains of different topologies, lengths, and functional consequences [48].

Significant progress has been made not only in understanding the function and important regulatory roles of the Ubl network but also the alterations of ubiquitination in cellular processes pertinent in the development of various human diseases including cancer [49]. This has led to the development of chemical and/or peptide molecules that inhibit components of the ubiquitination system [48], Bortezomib, the proteasome inhibitor, being the well known example [50]. Notably, E3 ligases that confer specificity of conjugation to substrate proteins and the deubiquitinating enzymes also have been extensively investigated as potential drug targets [51, 52]. In comparison, studies on the ubiquitin conjugation system as a source of potential drug targets in parasitic protozoa are very limited [53].

Studies on Ub in trypanosomatid parasites such as *T. brucei* and *T. cruzi* focused on revealing the Ub gene structure, Ub-dependent protein degradation, and its role in differentiation from the trypomastigote into an amastigote [54, 55]. Studies in *Plasmodium* identified deubiquitinating/deNeddylating activities and sumoylation of telomere associated protein PfSir2, a novel substrate protein for SUMO [56, 57]. Recent studies have demonstrated the role of ubiquitination in the degradation of transmembrane surface proteins in trypanosomes, cell cycle regulation by the single SUMO homologue in *T. brucei*, and interactions with several nuclear proteins in the host cell by a protein that possesses a ubiquitin ligase activity secreted by *T. cruzi*. [58, 59]. Further studies elucidating structural mechanisms of UCHL3, a hydrolase with uniquely dual specificities to Ub and NEDD in *Plasmodium*, further emphasize the increasing interest in parasitic Ub conjugation/deconjugation pathways as potential drug targets [60].

Studies in our laboratory with *Leishmania* Ufm1, a mitochondrial associated Ubl, revealed ways in which Ubl conjugation in these human parasites could represent novel protein drug targets [61]. The description of a Ubl (Ufm1), E1 enzyme (Uba5), and E2 enzyme (Ufc1) shows remarkable similarity of the *Leishmania* conjugation system to mammalian systems. This similarity suggests that anticancer drugs, for example, that target the ubiquitin pathway, may provide a starting point for development of effective antiparasitics. Yet, the sequence divergence of the *Leishmania* components from their mammalian homologues and the lack of similarity of Ufm1-conjugated target proteins to mammalian conjugates suggest that drugs can be developed avoiding toxic side effects. The antiparasitic effect of chemical disruption of this pathway is indicated by the reduced survival of intracellular amastigotes in which Ufm-1 function has been disrupted by overexpression of dominant negative mutant forms of Ufm1 or the E1 enzyme, Uba5 [61]. Identification of Ufm1-mediated protein modification pathways in *Leishmania*, with its distinct subset of substrate proteins associated with mitochondrial activities, may provide specific targets for novel drug therapies against this human pathogen.

The diversity of functions regulated by the Ubls in eukaryotic organisms in general and the fact that inhibitors of the ubiquitin-proteasome pathway are either in clinical use or are being studied for their potential as anticancer drugs indicate the importance of this pathway as a drug target. The ubiquitin-dependent proteolysis system (UPS) is increasingly recognized as a viable therapeutic pathway in the treatment of cancer after the successful treatment of hematological malignancies with proteasome inhibitors [62]. Deubiquitinases, the key effectors of UPS and intracellular signaling cascades, and Ub ligases because of their narrow substrate specificity are emerging as important targets for potential anticancer therapies. This effectiveness at stopping uncontrolled cancer cell growth suggests that targeting the ubiquitin pathways in human parasitic organisms may be successful as well. Importantly, the finding that protozoan parasites such as *Leishmania* interfere with the host protein degradation system to promote their intracellular survival [63] supports the concept that chemotherapy to reverse this interference could help clear the infection. Therefore, systematic studies of Ubl pathways in the human trypanosomatid parasites such as *Leishmania* could yield better understanding of the pathogenesis and lead to novel therapeutic reagents.

## 4. The Programmed Cell Death Pathway Presents Many Potential Targets for Antileishmanial Drug Therapy

Programmed cell death, commonly manifested as apoptosis, plays crucial roles in a multitude of physiological processes starting from embryogenesis to maintenance of the immune system. Evolutionarily, apoptosis emerged along with multicellular organisms, primarily as a defense against viral infections. However, increasing experimental evidence is showing that mechanistically similar processes also appear in many single-celled organisms including trypanosomatid parasites.

In trypanosomatids, features suggesting apoptosis have been reported in response to a wide range of stimuli such as heat shock, reactive oxygen species, antiparasitic drugs, prostaglandins, and antimicrobial peptides. Many biochemical events that accompany mammalian apoptosis such as generation of reactive oxygen species, increase in cytosolic Ca^2+^ levels, alterations in mitochondrial outer membrane potential, exposure of phosphatidylserine in the outer leaflet of the plasma membrane, release of cytochrome c and nucleases that cleave genomic DNA have also been widely documented in trypanosomatid parasites [64, 65].

In comparison to *C. elegans* and yeast, studies elucidating molecular mechanisms of PCD in trypanosomatid parasites are limited primarily because of the apparent absence of homologues to key regulatory or effector molecules of apoptosis in the trypanosomatid genomes that have been described in mammalian or nematode apoptosis such as Bcl-2 family members and caspases [66]. However, progress is being made with regard to systematic identification and characterization of proteases and/or nucleases with pro-apoptotic activities in these organisms [67]. We provided evidence that metacaspases (protease belonging to the caspase family) could be involved in *Leishmania* PCD [67]. Metacaspases have also been shown to be associated with cell cycle progression in *Leishmania *[68] and associated with RAB11-positive endosomes in *Trypanosoma brucei *[69] indicating additional roles not related to the cell death pathway. Several mammalian cell death regulators have additional functions in healthy cells and are not simply “latent” death factors waiting to kill cells [70]. A series of metacaspase inhibitors have been evaluated as potential antiparasitic drugs [71]. Recently, we and others have shown the involvement of mitochondrial nuclease endonuclease G in trypanosomatid PCD [72, 73]. The absence of homologues of regulatory or effector molecules of mammalian apoptosis indicates that the apoptotic pathways in these parasitic organisms are probably more austere/less complicated than in mammalian cells.

Although the impact of PCD pathways in regulating host-pathogen interaction in terms of parasite cell densities on the one hand and modulating host immune responses that favor the parasite on the other continues to be unraveled, the existence of conserved apoptotic cell death pathways in trypanosomatid parasites can provide targets for identifying novel chemotherapies [74]. Recent pharmacological studies elicited interest in several molecules with activities that trigger apoptotic death in cancerous cells as potential antiparasitic agents [75]. This is partly because of the common biochemical pathways used by the cancer cells and the parasites such as protein kinase pathways, DNA, and polyamine metabolism and also immune evasion strategies that underlie successful survival in the host.

Apoptotic death was observed in *Leishmania* treated with known antileishmanial drugs such as antimonial compounds [76] and antifungal compounds [64]. Antivirals, such as HIV-1 protease inhibitor Nelfinavir, induced oxidant stress-mediated apoptosis in *Leishmania *[77]. Cysteine cathepsin inhibitors have been shown to induce cell death in *Leishmania *[78]. Importantly, recent studies that characterized the action of novel drugs in *Leishmania* indicated that these drugs interfere and/or impair mitochondrial activities including an imbalance of antioxidant homeostasis [79–81]. There is indication that plant products such as yangambin and diospyrin induce apoptosis like death in *Leishmania* [82, 83]. Tafenoquines, an antimalarial compound, also induces apoptotic cell death in *Leishmania* by inhibiting mitochondrial cytochrome c reductase [84]. Fungal peptides with antitumoral activities kill *Leishmania* through apoptosis-like processes [85] involving depletion of ATP pools indicating impaired mitochondrial functions. Interestingly, overexpression of ascorbate peroxidase, a mitochondrial enzyme that scavenges reactive oxygen species in *Leishmania*, resulted in reduced cell death induced either by chemical agents or by reduced ATP generation [86].

Systematic characterization of programmed cell death pathways in trypanosomatid parasites could lead to identification of novel drug targets as it is evident that the human parasites utilize these pathways in unique ways for promoting infection [87]. In addition, such studies will be useful in defining the mechanism of action of novel drugs that induce apoptosis in these parasites. Several studies referenced above have shown apoptosis-like death in the parasites when treated with pharmacological compounds even though at present molecular mechanisms regulating such apoptotic death in trypanosomatid parasites are far from complete.

## 5. *Leishmania* Endoplasmic Reticulum Quality Control Molecules Involved in Secretion of Virulence Factors as Potential Targets for Novel Antileishmanial Drugs

*Leishmania* secrete a significant number of proteins into their environment that traffic through the secretory pathway (e.g., secretory acid phosphatase, chitinase, or thiol-specific antioxidant) [88–91]. Some of these secreted molecules have been shown to be important virulence factors involved in *Leishmania* pathogenesis. Although poorly studied, it is believed that secreted proteins traffic in *Leishmania* via a typical eukaryotic secretion pathway in which proteins are first folded in the ER and then transported via a Golgi apparatus to the flagellar reservoir for secretion outside the cell [92]. Therefore, the processing of putative virulence factors in the ER and their proper transport via the Golgi is essential for the survival of *Leishmania* parasites in their hosts.

A number of homologues of proteins involved in the quality control of glycoprotein folding of higher eukaryotes have been described in trypanosomatid parasites. These include calreticulin (CR), BiP, and protein disulfide isomerase (PDI) [93–95]. Our studies have focused on the characterization of CR and PDI and their possible involvement in the control of protein secretion in *L. donovani*. *L. donovani* calreticulin (LdCR) possesses the hallmarks of calreticulins, including its presence in the ER and conservation of protein structure suggesting conservation of function as a chaperone molecule [12]. The role as chaperone is indicated when altering the function of calreticulin affected the secretion of secretory acid phosphatases and resulted in significant decrease in survivability of *L. donovani* in human macrophages [12]. In addition, attempts to delete LdCR, a single-copy gene, in *L. donovani* were unsuccessful, only resulting in gene rearrangements [96]. Failure to generate a null mutant in *Leishmania* coupled with the absence of calnexin, a functional homolog of calreticulin, further suggests that LdCR plays an essential function in this organism. 

We have also shown that the *L. donovani* PDI (LdPDI) is a 12 kDa protein with a single domain containing the-CGHC-PDI signature [97]. That LdPDI has both oxidase and isomerase activities and is localized in the ER of *Leishmania* strongly suggests its role as an ER quality control enzyme responsible for disulfide bond formation in nascent polypeptides as described in higher eukaryotes [97]. The essential nature of PDIs was reported recently in mammalian cells by knocking down PDI in human breast cancer cells using small interfering RNAs [98]. PDI transcript depletion had a strong cytotoxic effect and triggered apoptosis in these cells.

Evidence that LdPDI could be involved in the control of protein secretion in the ER came from the analysis of mutant *Leishmania* parasites overexpressing mutated versions of this protein. Results showed that the secretion of the *Leishmania* secretory acid phosphatases was significantly reduced [12, 97]. 

The exact molecular mechanisms involved in altered trafficking and secretion of SAcP proteins in the two *Leishmania* mutants remain unclear. The proposed hypothesis for this effect is that the expression of either mutated/inactive chaperone has a dominant negative effect on the interaction of nascent glycoproteins with the native LdCR and LdPDI and with other folding molecules in the ER. 

As a drug target, disruption of LdCR or LdPDI function using a small molecule inhibition approach could result in a similar disruption of secretion. In that regard, a complete inhibition of parasite growth was observed when *Leishmania major* was incubated in vitro with 2 mM zinc bacitracin, a known PDI inhibitor, and disease progression was attenuated when zinc bacitracin was locally applied as an ointment on the parasite inoculation site in BALB/c mice [99]. 

The findings that disruption of CR and PDI alter the function of the secretory pathway, *Leishmania *parasites with disrupted CR showed reduced survival in macrophages, and the antiparasitic activity of a PDI inhibitor suggest that this pathway is well worth further exploration as a source of drug targets.

## 6. Conclusion

**Figure 1:**
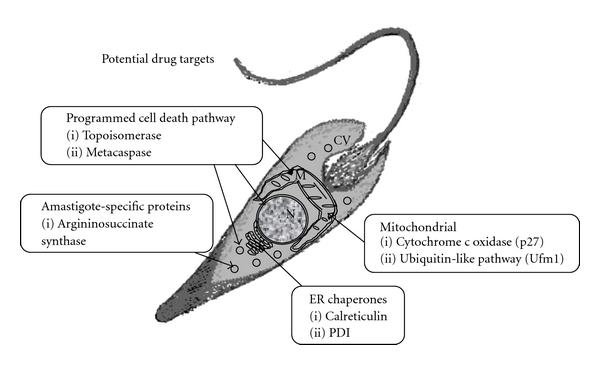
The potential drug targets discussed in this paper are listed, grouped according to the pathways and sites of action. N: nucleus, M: mitochondrion, CV: cytoplasmic vesicle, ER: endoplasmic reticulum, and PDI: protein disulfide isomerase.

The crucial need to develop new affordable drugs to cure leishmaniasis that can be delivered in a way that assures patient compliance and avoids rapid evolution of resistance on the part of this disfiguring and deadly parasite demands a multifaceted approach. Research to identify and characterize genes and the proteins they encode that are only known by untested homology or merely as hypothetical takes its place among others. High-throughput screening of off-the-shelf drugs and combinatorial libraries, repurposing of drugs with mechanisms that could suggest antiparasitic activity such as anticancer drugs and *in silico* approaches taking advantage of the annotated databases are all effective strategies in this multifaceted approach. In this paper, we have highlighted the important role that can be played by systematic molecular and cell biological studies of previously unknown genes and the proteins they encode to identify new drug targets and lay the bases for rational drug design (Figure 1).

## References

[1] Desjeux P (2004). Leishmaniasis: current situation and new perspectives. *Comparative Immunology, Microbiology and Infectious Diseases*.

[2] Herwaldt BL (1999). Leishmaniasis. *Lancet*.

[3] Handman E (2001). Leishmaniasis: current status of vaccine development. *Clinical Microbiology Reviews*.

[4] Gaskin AA, Schantz P, Jackson J (2002). Visceral leishmaniasis in a New York foxhound kennel. *Journal of Veterinary Internal Medicine*.

[5] Aronson N, Coleman R, Coyne P (2003). Cutaneous leishmaniasis in U.S. military personnel—southwest/central Asia, 2002-2003. *Morbidity and Mortality Weekly Report*.

[6] AABB (2003). Deferral for Risk of Leishmaniasis Exposure. *AABB Bulletin 03-14*.

[7] Palatnik-de-Sousa CB, Paraguai-de-Souza E, Gomes EM, Soares-Machado FC, Luz KG, Borojevic R (1996). Transmission of visceral leishmaniasis by blood transfusion in hamsters. *Brazilian Journal of Medical and Biological Research*.

[8] Giger U, Oakley DA, Owens SD, Schantz P (2002). Leishmania donovani transmission by packed RBC transfusion to anemic dogs in the United States. *Transfusion*.

[9] Molyneux D, Killick-Kendrick R, Peters W, Killick-Kendrick R (1987). Morphology, ultrastructure and life cycles. *The Leishmaniases in Biology and Medicine*.

[10] Goyard S, Segawa H, Gordon J (2003). An in vitro system for developmental and genetic studies of Leishmania donovani phosphoglycans. *Molecular and Biochemical Parasitology*.

[11] Debrabant A, Joshi MB, Pimenta PFP, Dwyer DM (2004). Generation of *Leishmania donovani* axenic amastigotes: their growth and biological characteristics. *International Journal for Parasitology*.

[12] Debrabant A, Lee N, Pogue GP, Dwyer DM, Nakhasi HL (2002). Expression of calreticulin P-domain results in impairment of secretory pathway in Leishmania donovani and reduced parasite survival in macrophages. *International Journal for Parasitology*.

[13] Selvapandiyan A, Debrabant A, Duncan R (2004). Centrin gene disruption impairs stage-specific basal body duplication and cell cycle progression in Leishmania. *Journal of Biological Chemistry*.

[14] Croft SL, Coombs GH (2003). Leishmaniasis—current chemotherapy and recent advances in the search for novel drugs. *Trends in Parasitology*.

[15] Davis AJ, Murray HW, Handman E (2004). Drugs against leishmaniasis: a synergy of technology and partnerships. *Trends in Parasitology*.

[16] Dujardin JC, González-Pacanowska D, Croft SL, Olesen OF, Späth GF (2010). Collaborative actions in anti-trypanosomatid chemotherapy with partners from disease endemic areas. *Trends in Parasitology*.

[17] Myler PJ, Sisk E, McDonagh PD (2000). Genomic organization and gene function in Leishmania. *Biochemical Society Transactions*.

[18] Crowther GJ, Shanmugam D, Carmona SJ (2010). Identification of attractive drug targets in neglected-disease pathogens using an in Silico approach. *PLoS Neglected Tropical Diseases*.

[19] McConville MJ, Handman E (2007). The molecular basis of Leishmania pathogenesis. *International Journal for Parasitology*.

[20] Naderer T, McConville MJ (2008). The Leishmania-macrophage interaction: a metabolic perspective. *Cellular Microbiology*.

[21] McConville MJ, de Souza D, Saunders E, Likic VA, Naderer T (2007). Living in a phagolysosome; metabolism of Leishmania amastigotes. *Trends in Parasitology*.

[22] Hart DT, Vickerman K, Coombs GH (1981). Respiration of Leishmania mexicana amastigotes and promastigotes. *Molecular and Biochemical Parasitology*.

[23] Van Hellemond JJ, Tielens AGM (1997). Inhibition of the respiratory chain results in a reversible metabolic arrest in Leishmania promastigotes. *Molecular and Biochemical Parasitology*.

[24] Santhamma KR, Bhaduri A (1995). Characterization of the respiratory chain of Leishmania donovani promastigotes. *Molecular and Biochemical Parasitology*.

[25] Van Hellemond JJ, Bakker BM, Tielens AGM (2005). Energy metabolism and its compartmentation in Trypanosoma brucei. *Advances in Microbial Physiology*.

[26] Luque-Ortega JR, Rivas L (2007). Miltefosine (hexadecylphosphocholine) inhibits cytochrome c oxidase in Leishmania donovani promastigotes. *Antimicrobial Agents and Chemotherapy*.

[27] Saugar JM, Delgado J, Hornillos V (2007). Synthesis and biological evaluation of fluorescent leishmanicidal analogues of hexadecylphosphocholine (Miltefosine) as probes of antiparasite mechanisms. *Journal of Medicinal Chemistry*.

[28] Speijer D, Breek CKD, Muijsers AO (1996). The sequence of a small subunit of cytochrome c oxidase from Crithidia fasciculata which is homologous to mammalian subunit IV. *FEBS Letters*.

[29] Horváth A, Berry EA, Huang LS, Maslov DA (2000). Leishmania tarentolae: a parallel isolation of cytochrome bc_1_ and cytochrome c oxidase. *Experimental Parasitology*.

[30] Horváth A, Kingan TG, Maslov DA (2000). Detection of the mitochondrially encoded cytochrome c oxidase subunit I in the trypanosomatid protozoan Leishmania tarentolae: evidence for translation of unedited mRNA in the kinetoplast. *Journal of Biological Chemistry*.

[31] Horváth A, Horáková E, Dunajčíková P (2005). Downregulation of the nuclear-encoded subunits of the complexes III and IV disrupts their respective complexes but not complex I in procyclic Trypanosoma brucei. *Molecular Microbiology*.

[32] Uboldi AD, Lueder FB, Walsh P (2006). A mitochondrial protein affects cell morphology, mitochondrial segregation and virulence in Leishmania. *International Journal for Parasitology*.

[33] Zíková A, Panigrahi AK, Uboldi AD, Dalley RA, Handman E, Stuart K (2008). Structural and functional association of Trypanosoma brucei MIX protein with cytochrome c oxidase complex. *Eukaryotic Cell*.

[34] Dey R, Meneses C, Salotra P, Kamhawi S, Nakhasi HL, Duncan R (2010). Characterization of a Leishmania stage-specific mitochondrial membrane protein that enhances the activity of cytochrome c oxidase and its role in virulence. *Molecular Microbiology*.

[35] Krungkrai J, Krungkrai SR, Suraveratum N, Prapunwattana P (1997). Mitochondrial ubiquinol-cytochrome C reductase and cytochrome C oxidase: chemotherapeutic targets in malarial parasites. *Biochemistry and Molecular Biology International*.

[36] Zhao Y, Hanton WK, Lee KH (1986). Antimalarial agents, 2. Artesunate, an inhibitor of cytochrome oxidase activity in Plasmodium berghei. *Journal of Natural Products*.

[37] Krungkrai J (2004). The multiple roles of the mitochondrion of the malarial parasite. *Parasitology*.

[38] Duncan R, Dey R, Tomioka K, Hairston H, Selvapandiyan A, Nakhasi HL (2009). Biomarkers of attenuation in the Leishmania donovani centrin gene deleted cell line-requirements for safety in a live vaccine candidate. *The Open Parasitology Journal*.

[39] Haines RJ, Pendleton LC, Eichler DC (2011). Argininosuccinate synthase: at the center of arginine metabolism. *International Journal of Biochemistry and Molecular Biology*.

[40] Husson A, Brasse-Lagnel C, Fairand A, Renouf S, Lavoinne A (2003). Argininosuccinate synthetase from the urea cycle to the citrulline-NO cycle. *European Journal of Biochemistry*.

[41] Opperdoes FR, Szikora JP (2006). In silico prediction of the glycosomal enzymes of Leishmania major and trypanosomes. *Molecular and Biochemical Parasitology*.

[42] Lakhal-Naouar I, Nakhasi HL, Duncan R Characterization of the *Leishmania donovani* Argininosuccinate Synthase.

[43] Shukla AK, Singh BK, Patra S, Dubey VK (2010). Rational approaches for drug designing against leishmaniasis. *Applied Biochemistry and Biotechnology*.

[44] Jenkins GR, Tolleson WH, Newkirk DK (2000). Identification of fumonisin B_1_ as an inhibitor of argininosuccinate synthetase using fumonisin affinity chromatography and in vitro kinetic studies. *Journal of Biochemical and Molecular Toxicology*.

[45] Ameen M, Palmer T (1987). Inhibition of urea cycle enzymes by lysine and saccharopine. *Biochemistry International*.

[46] Hershko A, Ciechanover A (1998). The ubiquitin system. *The Annual Review of Biochemistry*.

[47] Hochstrasser M (2009). Origin and function of ubiquitin-like proteins. *Nature*.

[48] Hoeller D, Dikic I (2009). Targeting the ubiquitin system in cancer therapy. *Nature*.

[49] Ande SR, Chen J, Maddika S (2009). The ubiquitin pathway: an emerging drug target in cancer therapy. *European Journal of Pharmacology*.

[50] Adams J (2004). The development of proteasome inhibitors as anticancer drugs. *Cancer Cell*.

[51] Goldenberg SJ, Marblestone JG, Mattern MR, Nicholson B (2010). Strategies for the identification of ubiquitin ligase inhibitors. *Biochemical Society Transactions*.

[52] Sacco JJ, Coulson JM, Clague MJ, Urbé S (2010). Emerging roles of deubiquitinases in cancer-associated pathways. *IUBMB Life*.

[53] Ponder EL, Bogyo M (2007). Ubiquitin-like modifiers and their deconjugating enzymes in medically important parasitic protozoa. *Eukaryotic Cell*.

[54] Kirchhoff LV, Kim KS, Engman DM, Donelson JE (1988). Ubiquitin genes in trypanosomatidae. *Journal of Biological Chemistry*.

[55] Fleischmann J, Campbell DA (1994). Expression of the Leishmania tarentolae ubiquitin-encoding and mini-exon genes. *Gene*.

[56] Artavanis-Tsakonas K, Misaghi S, Comeaux CA (2006). Identification by functional proteomics of a deubiquitinating/deNeddylating enzyme in Plasmodium falciparum. *Molecular Microbiology*.

[57] Issar N, Roux E, Mattei D, Scherf A (2008). Identification of a novel post-translational modification in Plasmodium falciparum: protein sumoylation in different cellular compartments. *Cellular Microbiology*.

[58] Chung WL, Leung KF, Carrington M, Field MC (2008). Ubiquitylation is required for degradation of transmembrane surface proteins in Trypanosomes. *Traffic*.

[59] Hashimoto M, Murata E, Aoki T (2010). Secretory protein with RING finger domain (SPRING) specific to Trypanosoma cruzi is directed, as a ubiquitin ligase related protein, to the nucleus of host cells. *Cellular Microbiology*.

[60] Artavanis-Tsakonas K, Weihofen WA, Antos JM (2010). Characterization and structural studies of the Plasmodium falciparum ubiquitin and Nedd8 hydrolase UCHL3. *Journal of Biological Chemistry*.

[61] Gannavaram S, Sharma P, Duncan RC, Salotra P, Nakhasi HL (2011). Mitochondrial associated ubiquitin fold modifier-1 mediated protein conjugation in Leishmania donovani. *PLoS ONE*.

[62] Eldridge AG, O'Brien T (2010). Therapeutic strategies within the ubiquitin proteasome system. *Cell Death and Differentiation*.

[63] Olivier M, Gregory DJ, Forget G (2005). Subversion mechanisms by which Leishmania parasites can escape the host immune response: a signaling point of view. *Clinical Microbiology Reviews*.

[64] Lee N, Bertholet S, Debrabant A, Muller J, Duncan R, Nakhasi HL (2002). Programmed cell death in the unicellular protozoan parasite Leishmania. *Cell Death and Differentiation*.

[65] van Zandbergen G, Lüder CGK, Heussler V, Duszenko M (2010). Programmed cell death in unicellular parasites: a prerequisite for sustained infection?. *Trends in Parasitology*.

[66] Smirlis D, Duszenko M, Ruiz AJ (2010). Targeting essential pathways in trypanosomatids gives insights into protozoan mechanisms of cell death. *Parasites and Vectors*.

[67] Lee N, Gannavaram S, Selvapandiyan A, Debrabant A (2007). Characterization of metacaspases with trypsin-like activity and their putative role in programmed cell death in the protozoan parasite Leishmania. *Eukaryotic Cell*.

[68] Ambit A, Fasel N, Coombs GH, Mottram JC (2008). An essential role for the Leishmania major metacaspase in cell cycle progression. *Cell Death and Differentiation*.

[69] Helms MJ, Ambit A, Appleton P, Tetley L, Coombs GH, Mottram JC (2006). Bloodstream form Trypanosoma brucei depend upon multiple metacaspases associated with RAB11-positive endosomes. *Journal of Cell Science*.

[70] Cheng WC, Berman SB, Ivanovska I (2006). Mitochondrial factors with dual roles in death and survival. *Oncogene*.

[71] Berg M, Van der Veken P, Joossens J (2010). Design and evaluation of Trypanosoma brucei metacaspase inhibitors. *Bioorganic and Medicinal Chemistry Letters*.

[72] Gannavaram S, Vedvyas C, Debrabant A (2008). Conservation of the pro-apoptotic nuclease activity of endonuclease G in unicellular trypanosomatid parasites. *Journal of Cell Science*.

[73] Rico E, Alzate JF, Arias AA (2009). Leishmania infantum expresses a mitochondrial nuclease homologous to EndoG that migrates to the nucleus in response to an apoptotic stimulus. *Molecular and Biochemical Parasitology*.

[74] Lüder CG, Campos-Salinas J, Gonzalez-Rey E, Van Zandbergen G (2010). Impact of protozoan cell death on parasite-host interactions and pathogenesis. *Parasites and Vectors*.

[75] Fuertes MA, Nguewa PA, Castilla J, Alonso C, Pérez JM (2008). Anticancer compounds as leishmanicidal drugs: challenges in chemotherapy and future perspectives. *Current Medicinal Chemistry*.

[76] Vergnes B, Gourbal B, Girard I, Sundar S, Drummelsmith J, Ouellette M (2007). A proteomics screen implicates HSP83 and a small kinetoplastid calpain-related protein in drug resistance in Leishmania donovani clinical field isolates by modulating drug-induced programmed cell death. *Molecular and Cellular Proteomics*.

[77] Kumar P, Lodge R, Trudel N, Ouellet M, Ouellette M, Tremblay MJ (2010). Nelfinavir, an HIV-1 protease inhibitor, induces oxidative stress-mediated, caspase-independent apoptosis in Leishmania amastigotes. *PLoS Neglected Tropical Diseases*.

[78] Schurigt U, Schad C, Glowa C (2010). Aziridine-2,3-dicarboxylate-based cysteine cathepsin inhibitors induce cell death in Leishmania major associated with accumulation of debris in autophagy-related lysosome-like vacuoles. *Antimicrobial Agents and Chemotherapy*.

[79] Delorenzi JC, Attias M, Gattass CR (2001). Antileishmanial activity of an indole alkaloid from Peschiera australis. *Antimicrobial Agents and Chemotherapy*.

[80] Borges VM, Lopes UG, De Souza W, Vannier-Santos MA (2005). Cell structure and cytokinesis alterations in multidrug-resistant Leishmania (Leishmania) amazonensis. *Parasitology Research*.

[81] Ueda-Nakamura T, Mendonç-Filho RR, Morgado-Díaz JA (2006). Antileishmanial activity of Eugenol-rich essential oil from Ocimum gratissimum. *Parasitology International*.

[82] Mukherjee P, Majee SB, Ghosh S, Hazra B (2009). Apoptosis-like death in Leishmania donovani promastigotes induced by diospyrin and its ethanolamine derivative. *International Journal of Antimicrobial Agents*.

[83] Neto RLM, Sousa LMA, Dias CS, Filho JMB, Oliveira MR, Figueiredo RCB (2011). Morphological and physiological changes in Leishmania promastigotes induced by yangambin, a lignan obtained from Ocotea duckei. *Experimental Parasitology*.

[84] Carvalho L, Luque-Ortega JR, Manzano JI, Castanys S, Rivas L, Gamarro F (2010). Tafenoquine, an antiplasmodial 8-aminoquinoline, targets Leishmania respiratory complex III and induces apoptosis. *Antimicrobial Agents and Chemotherapy*.

[85] Luque-Ortega JR, Cruz LJ, Albericio F, Rivas L (2010). The antitumoral depsipeptide IB-01212 kills Leishmania through an apoptosis-like process involving intracellular targets. *Molecular Pharmaceutics*.

[86] Dolai S, Yadav RK, Pal S, Adak S (2009). Overexpression of mitochondrial Leishmania major ascorbate peroxidase enhances tolerance to oxidative stress-induced programmed cell death and protein damage. *Eukaryotic Cell*.

[87] Laskay T, van Zandbergen G, Solbach W (2008). Neutrophil granulocytes as host cells and transport vehicles for intracellular pathogens: apoptosis as infection-promoting factor. *Immunobiology*.

[88] Bates PA, Dwyer DM (1987). Biosynthesis and secretion of acid phosphatase by Leishmania donovani promastigotes. *Molecular and Biochemical Parasitology*.

[89] Shakarian AM, Dwyer DM (1998). The Ld Cht1 gene encodes the secretory chitinase of the human pathogen Leishmania donovani. *Gene*.

[90] Webb JR, Campos-Neto A, Ovendale PJ (1998). Human and murine immune responses to a novel Leishmania major recombinant protein encoded by members of a multicopy gene family. *Infection and Immunity*.

[91] Silverman JM, Chan SK, Robinson DP (2008). Proteomic analysis of the secretome of Leishmania donovani. *Genome Biology*.

[92] McConville MJ, Mullin KA, Ilgoutz SC, Teasdale RD (2002). Secretory pathway of trypanosomatid parasites. *Microbiology and Molecular Biology Reviews*.

[93] Bangs JD, Brouch EM, Ransom DM, Roggy JL (1996). A soluble secretory reporter system in Trypanosoma brucei. Studies on endoplasmic reticulum targeting. *Journal of Biological Chemistry*.

[94] Joshi M, Pogue GP, Duncan RC (1996). Isolation and characterization of *Leishmania donovani* calreticulin gene and its conservation of the RNA binding activity. *Molecular and Biochemical Parasitology*.

[95] Hong BX, Soong L (2008). Identification and enzymatic activities of four protein disulfide isomerase (PDI) isoforms of Leishmania amazonensis. *Parasitology Research*.

[96] Debrabant A, Nakhasi HL Genetic manipulation of the calreticulin gene in *Leishmania donovani*.

[97] Padilla A, Noiva R, Lee N, Mohan KVK, Nakhasi HL, Debrabant A (2003). An atypical protein disulfide isomerase from the protozoan parasite Leishmania containing a single thioredoxin-like domain. *Journal of Biological Chemistry*.

[98] Hashida T, Kotake Y, Ohta S (2011). Protein disulfide isomerase knockdown-induced cell death is cell-line-dependent and involves apoptosis in MCF-7 cells. *Journal of Toxicological Sciences*.

[99] Ben Achour Y, Chenik M, Louzir H, Dellagi K (2002). Identification of a disulfide isomerase protein of Leishmania major as a putative virulence factor. *Infection and Immunity*.

